# Manipulation of free-floating objects using Faraday flows and deep reinforcement learning

**DOI:** 10.1038/s41598-021-04204-9

**Published:** 2022-01-10

**Authors:** David Hardman, Thomas George Thuruthel, Fumiya Iida

**Affiliations:** Bio-Inspired Robotics Lab, Department of Engineering, University of Cambridge, Cambridge, CB2 1PZ UK

**Keywords:** Fluid dynamics, Statistical physics, thermodynamics and nonlinear dynamics, Computational science

## Abstract

The ability to remotely control a free-floating object through surface flows on a fluid medium can facilitate numerous applications. Current studies on this problem have been limited to uni-directional motion control due to the challenging nature of the control problem. Analytical modelling of the object dynamics is difficult due to the high-dimensionality and mixing of the surface flows while the control problem is hard due to the nonlinear slow dynamics of the fluid medium, underactuation, and chaotic regions. This study presents a methodology for manipulation of free-floating objects using large-scale physical experimentation and recent advances in deep reinforcement learning. We demonstrate our methodology through the open-loop control of a free-floating object in water using a robotic arm. Our learned control policy is relatively quick to obtain, highly data efficient, and easily scalable to a higher-dimensional parameter space and/or experimental scenarios. Our results show the potential of data-driven approaches for solving and analyzing highly complex nonlinear control problems.

## Introduction

Remote control of floating objects on a fluid surface has numerous applications ranging from manipulation of micro-particles^1–3^ to macro-particles^4^. This is usually performed by transfer of energy through the surrounding medium^1,4,5^ or through power sources that can be localized remotely such as magnetic fields and optical forces^6–8^. Controlled manipulation through the surrounding medium is highly desirable as it is potentially more scalable to larger areas and varying objects using simple control sources. The control objective is to generate surface flows on the fluid surface by parametrically excited high-dimensional local wave sources. These create two-dimensional horizontal velocity fields at the surface, also known as Faraday flows, that have attributes of two-dimensional turbulence; characterised by surface vortices and local Lagrangian coherent structures^4,9–11^. The study of these surface flows and their use for object transport has been limited due to difficulties in modelling this nonlinear system and the high-dimensionality of the environment. Hence, current work has been limited to empirical studies on uni-directional control of floating objects by one-dimensional wave sources^4^. Surface flows generated by multi-periodic wave sources are even limited to harmonic periods^12,13^. This work extends the state-of-the-art to the two-dimensional control of a floating object in water driven by multi-periodic wave sources using recent advances in deep reinforcement learning (DRL).

Developing optimal controllers directly from real-world experiments is appealing in fluid mechanics problems due to the difficulties in developing accurate mathematical models for them^14^. However, such controllers should be able to handle the high nonlinearity, high-dimensionality, non-convexity and underactuated nature of the problem. Deep learning-based approaches^15^ have been exceptional in handling the nonlinearity and dimensionality of the problem without falling into local minima^16–18^. Transferring these deep architectures to reinforcement learning has also shown promise in solving optimal control and design problems, particularly in shape optimization^19,20^ and flow control^21,22^. DRL was also used to control a rigid object using directed fluid jets in an animation game^23^. Nevertheless, the application of DRL for fluid control problems is still limited to simulation studies, anticlimactic to its actual strengths^21^.The few experimental works on this topic have explored control strategies for drag reduction on a bluff body^24^, dynamic control of microfluidic devices^25^ and flow separation control over an airfoil^26^.

**Figure 1:**
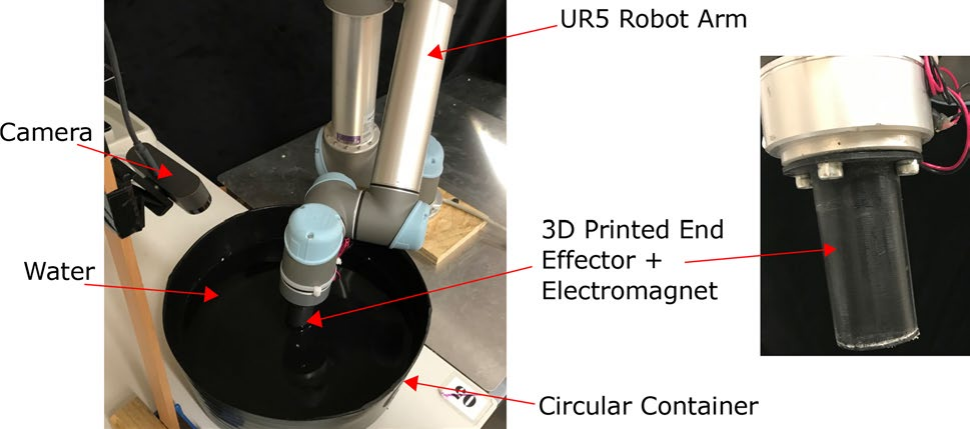
Experimental setup.

**Figure 2:**
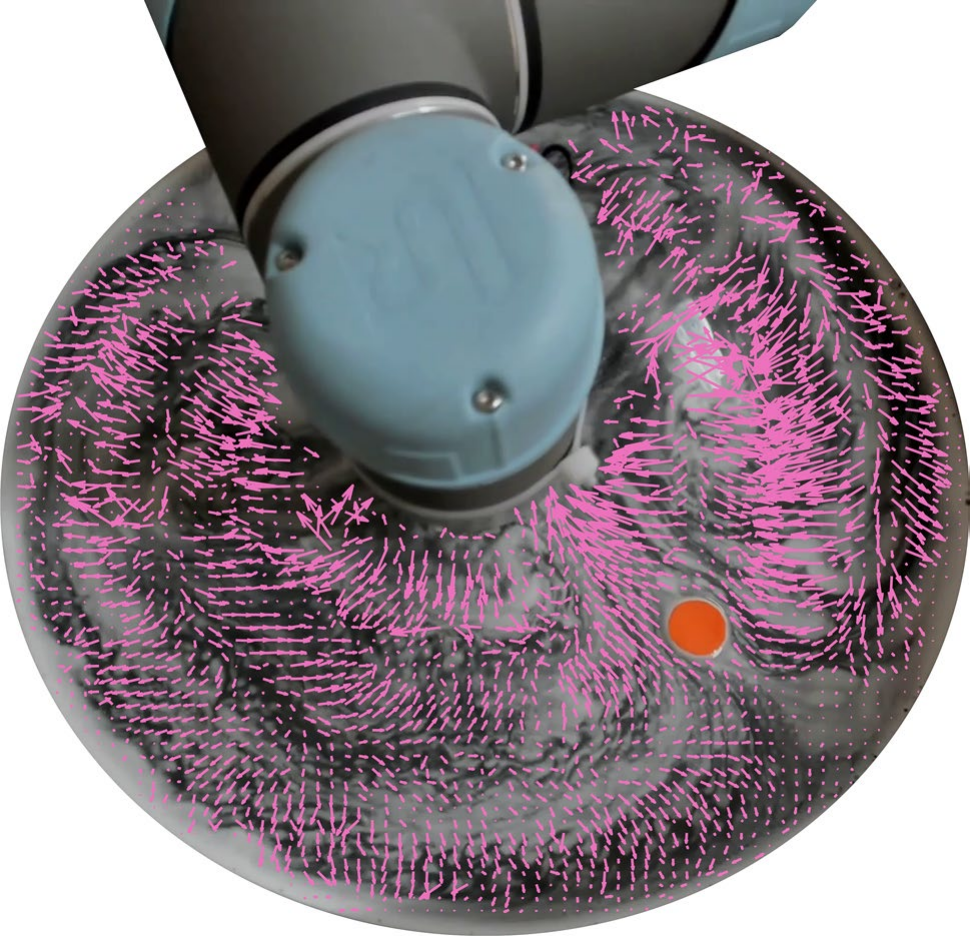
Manipulation of a free-floating object (in orange) by generating surface flows with a robotic arm. The free-floating object is guided across the water by the visualized surface flows.

This work presents the use of DRL for solving the problem of manipulating a free-floating object through surface flows. We structure the problem as an open-loop delivery task: given a randomized starting position of a floating object in a water tub, the controller must drive the object to a target location at the edge of the tub. The object is driven by the complex multi-period oscillation of a robotic arm submerged at the centre of the container (See Fig. 1). We assume that the problem can be described as a Markov Decision Process i.e. given the starting conditions of the floating object and a control sequence, the final position of the object can be represented by a probability distribution. The motion of the floating object emerges from its interactions with surface flows generated in the fluid; a typical flow pattern is visualized in Fig. 2. The motion of the arm is governed by 11 optimizable parameters (an average depth, 3 amplitudes, 3 frequencies, 2 phases, and 2 angles), which define oscillations in a small region about the tub’s centre (see Table 1 and Eq. 2). The controller receives no feedback about the object’s position until the end of each iteration, when a recorded camera feed is used to evaluate each attempt’s success. By restricting our actions to an open-loop case the physical setup is kept simple, requiring little sensory feedback, and lending itself to applications where delicate closed-loop systems are undesirable and/or impractical. After each iteration, an electromagnet is used to reset the object to a new starting position—see *Experimental Setup* and Supplementary Video 1.

In order to validate the selection of the 11 control parameters and their ranges, and to gain an insight into the key parameters necessary for DRL reward shaping, we first perform a global optimization routine for three selected tasks. Bayesian optimization (BO) is chosen for this preliminary investigation due to the high temporal cost of function evaluation, the relatively small number of control parameters, and the probabilistic nature of the problem itself^27^. In addition, the method does not require derivatives to be calculated, using only the evaluated value to update its prior.

Following this, a DRL algorithm—deep deterministic policy gradient (DDPG)—is used to learn the global controller for all tasks^28^. DDPG uses an off-policy deep actor-critic architecture to learn control policies for continuous action spaces. Given the cost of physical function evaluation, DDPG is beneficial for its concurrent learning of the value function and the policy, enabling it to benefit from both successful and unsuccessful trials; the actor learns to propose suitable constant values for each of the 11 parameters, whilst the critic uses previous attempts to predict the reward that these values will achieve. With this functionality, a global controller can be developed over a period of days using only data which has been physically collected. Details of the the BO and DDPG implementations are given in *Methods*, and in Supplementary Section S1.

Our experimental results show that even with the open-loop constraint and the nonlinear surface flows, the control problem can be solved with reasonable accuracy. Using empirical studies we investigate the complexity of the problem and the strategy behind the optimal control solutions. We show that this global control policy can also be obtained through a few days of training with reward shaping, even with the deep architectures we employ for our controller. Our results show how Lagrangian coherent structures in the surface flows can be used to generate repeatable trajectories. We also report the generation of quasi-periodic surface flows and transiently steady flows based on the target location. As the motion of the floating object is determined by the surface flows, we also show that the control policies are potentially transferable to objects of various shapes and sizes.

## Results

In order to study the feasibility and repeatability of the delivery task, we first use Bayesian optimization to confirm that the chosen ranges of the 11 control parameters are capable of performing three tasks: from a fixed starting position, the free-floating object must be driven to the container’s edge after rotating $0^{\circ }$, $90^{\circ }$, and $180^{\circ }$. Capillary forces are found to retain the floating object at the container’s edge once it first makes contact, such that the boundary $r = r_{container}$ behaves as a stable outer attractor.

**Figure 3:**
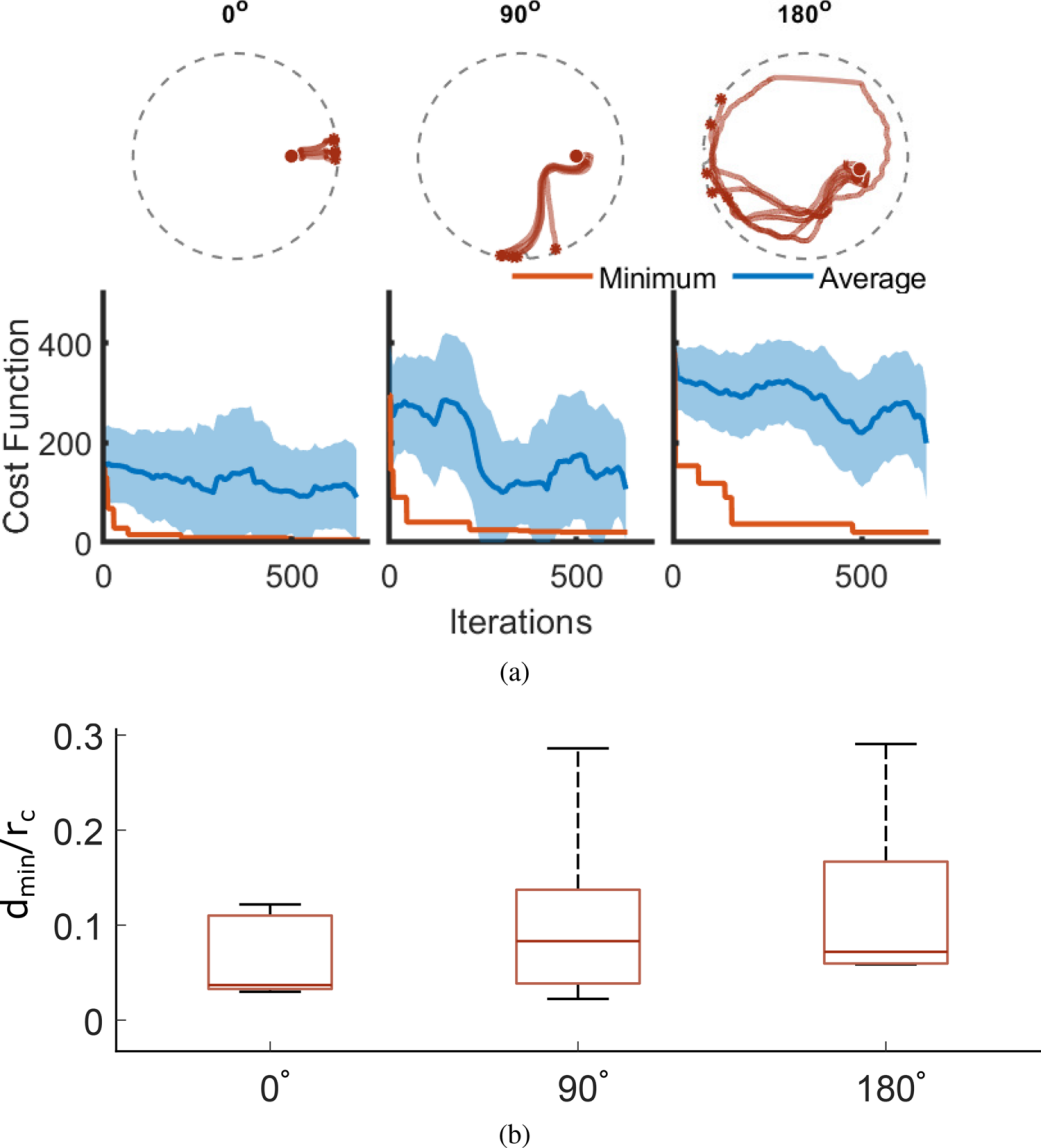
(**a**) Bayesian optimization results for the $0^{\circ }$, $90^{\circ }$, and $180^{\circ }$ open-loop delivery tasks. After 650 iterations, the best set of actions are repeated 5 times and the object’s path plotted. (**b**) Min/max boxplots of the floating object’s minimum distance from the target location—$d_{min}$—during the 5 repetitions,normalized by the container radius $r_c$.

For each, a separate Bayesian optimization is run to find a set of control parameters which minimize both the total time taken and the object’s final proximity to the target location (Eq. 4). Figure 3(a) shows the progress of the three optimizations alongside 5 repetitions of the best actions after 650 iterations (Supplementary Videos 2 and 3). Within the first 100 iterations low cost actions are found for the $0^{\circ }$ case and significantly improved upon, leading towards a highly repeatable method at the end of the optimization. Though the $90^{\circ }$ case takes longer to converge to a solution, a successful set of actions is still developed: only one of the 5 repetitions significantly deviates from the average path, but still reaches the edge close to the target location. We see more variance in the $180^{\circ }$ paths: though all of the repetitions come close to the target, none hit it directly, with one attempt never reaching the tub’s edge during the allotted time. The supplementary videos show the floating object’s smooth motion along the attractive Lagrangian coherent structures generated by the optimizations—despite small discrepancies in initial position, all repetitions follow qualitatively similar trajectories. Figure 3(b) compares the object’s minimum distance from the target location for each repetition: in all cases, this does not exceed 53 mm, suggesting that the delivery task is at least partially solvable within the chosen parameter ranges. Strong Lagrangian coherent structures develop around the object’s starting point, ensuring that the initial trajectories follow similar paths, imparting enough momentum to carry the object through regions in which the underlying flow is more chaotic (Supplementary Figures S11a and S11b). The $180^{\circ }$ case’s spread of $\theta$ values at which the edge is reached arises from the paths being, on average, longer than those of the $0^{\circ }$ and $90^{\circ }$ cases, giving small discrepancies in starting position longer time to diverge before nearing their stable attractor. The subsequent large spread of trajectories is visualized in Supplementary Figure S13a, in which dynamic time warping is used to evaluate the similarity of repeated paths. This trade-off between unrepeatabilities of the trajectory and the target angles is also observed with the DDPG controller, implying that this is a fundamental limitation of the system itself.

The Bayesian optimization results provide an insight into the complexity of the delivery tasks, helping to shape the reward functions for future DDPG attempts. As expected, driving the object directly away from the oscillating end-effector is an easier solution to find and is more repeatable. As the angle and path length increase, the observed trajectory becomes more unpredictable and hence more difficult to obtain through search algorithms. The Bayesian optimization takes $\sim$500 iterations to guarantee a good solution for each task which, with an upper bound of two minutes for each control episode, amounts to approximately 16 hours of real-world experiments. Generating global controllers using thousands of these local solutions is hence infeasible for practical purposes. DDPG, however, should be able to exploit any structure in the state space and control solutions to greatly reduce the physical data required, enabling a controller to be developed within a reasonable time frame.

**Figure 4:**
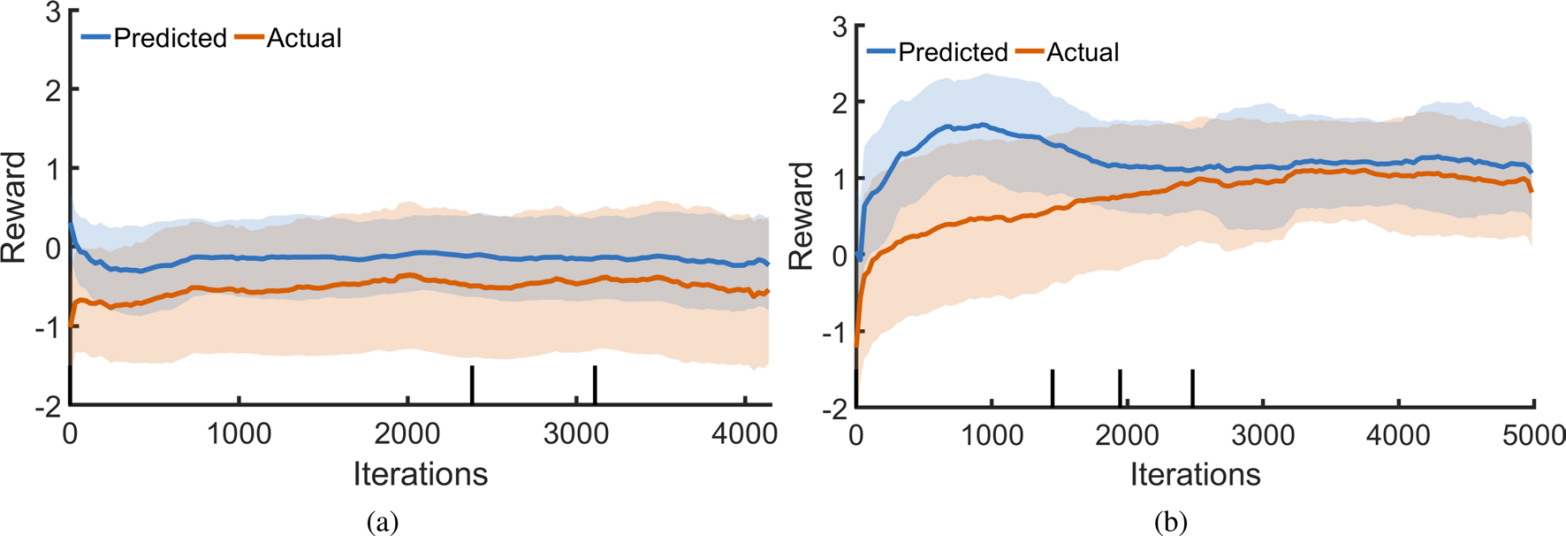
(**a**) DDPG training using a naïve reward function that tries to minimise the distance between target and object. (**b**) DDPG training with rewards shaped using prior knowledge.

For our study two DDPG agents are trained and analyzed. The first agent (*a*) uses a naïve reward function (Eqs. 5 and 7) that is concerned only with minimising the distance between the object’s centre and the target location. Though this implementation is straightforward, it is highly inefficient, with its training progress in Fig. 4(a) showing little improvement over 4000 iterations (see *Methods* and Supplementary Figure S1) for details.

The second agent (*b*) uses a reward function (Eqs. 6a and 6b) shaped by observations made during the BO tests. Primarily, we observe that it is easier to move radially than angularly, and that longer paths to the target location are considerably more sensitive to small changes in the initial position and the control parameters. To counter this, the reward is shaped such that early outward motions are encouraged, rewarding solutions which hit the edge much more highly than those which avoid it. This leads to a sharp rise in reward near the start of training—Fig. 4(b)—as the controller learns to find these high-reward actions. From here, the average reward rises gradually, as the average error between final and target location on the container’s edge is reduced. In addition, actions are rewarded for reaching the edge in short time, discouraging excessively long paths that are prone to non-repeatability. The resulting action spaces and predicted rewards for both trained agents are illustrated in Supplementary Figure S6.

This reward shaping technique allows the controller to develop a global control policy in a relatively short time using only the results of physical experiments. Not only are we able to obtain a higher reward faster, but the critic converges more rapidly for reward function *b*. For all further tests and analysis, agent *b* is selected due to its better performance.

**Figure 5:**
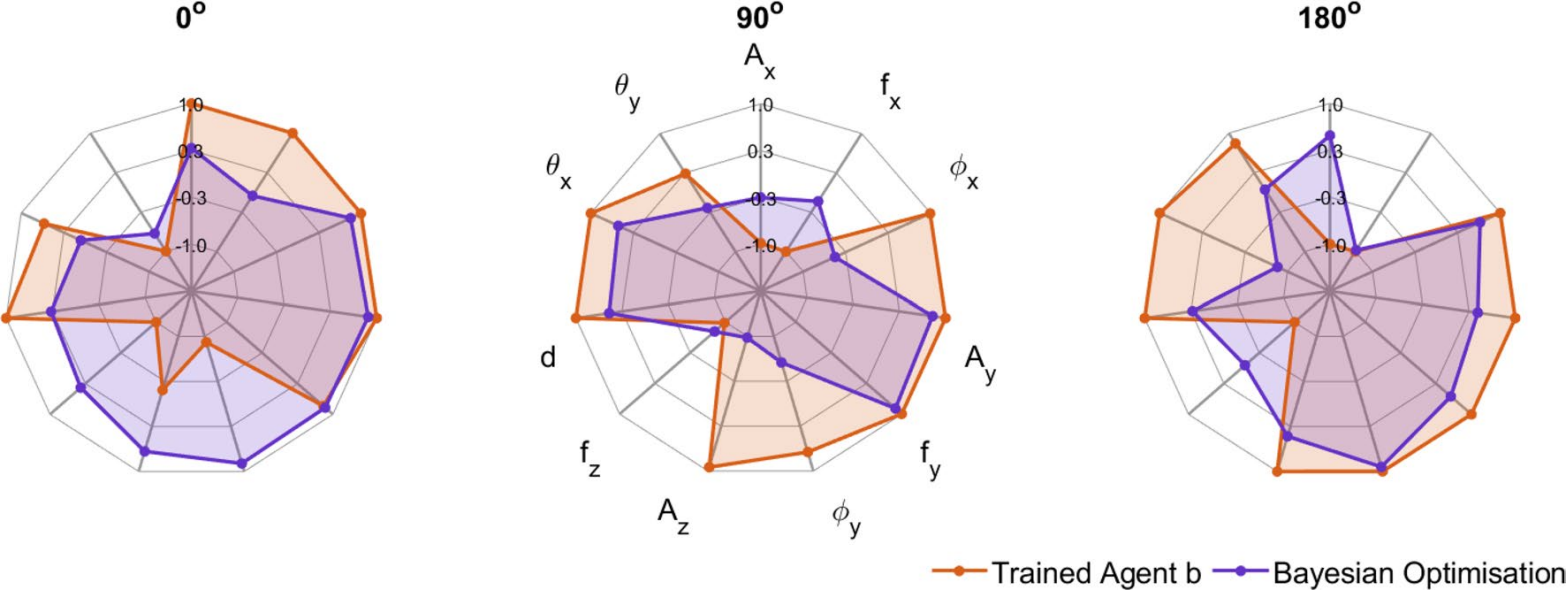
The proposed action spaces for $0^{\circ }$, $90^{\circ }$, and $180^{\circ }$ targets. Each of the control parameters is normalized in the range (−1,1).

Figure 5 compares the 11 control parameters proposed by the trained DDPG agents with those developed during the three Bayesian optimization tasks. The different patterns which can be observed between the two parameter sets demonstrate the non-uniqueness of the solutions. The DDPG solutions tend to be nearer the edges of the parameter ranges. With extensive training times these solutions would be expected to converge to a more optimal result: we next analyse the success of the proposed solutions after just a few days of training.

**Figure 6:**
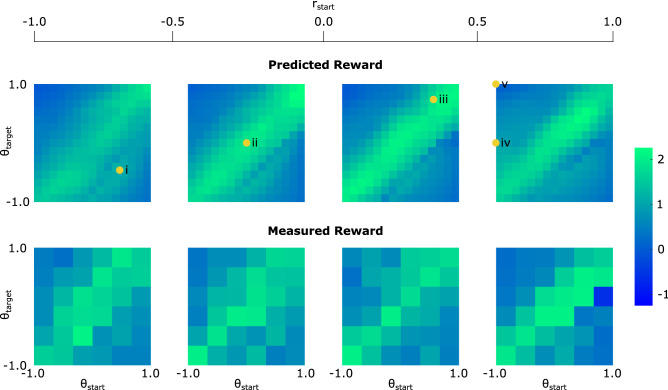
Predicted and measured rewards of the trained actor over the three dimensional start/target space, normalized over the range (−1, 1).

Due to the actor-critic architecture of DDPG, it is also possible to estimate the expected reward (Eqs. 6a and 6b) of a given action using the trained critic. The upper row of Fig. 6 plots these expected rewards over the entire 3D state-space, defined using the starting radius, starting angle, and target angle. Each is normalized over the range (−1, 1), as discussed in the *Control Space* section in *Methods*. The starting radius is incremented between columns. Regardless of this radius, strong diagonal bands demonstrate the ease with which the controller learns to push the object radially with high repeatability. As the angles separate, the predicted reward decreases, reflecting the increased problem complexities encountered in Fig. 3(a). Only two cases are predicted negative rewards—the upper left cells of the first and third plots which require $180^{\circ }$ rotations, though the predictions are still significantly higher than the minimum reward of −1.25. Interestingly, despite the radial symmetry of the setup, the lower right quadrants of the plots contain marginally higher rewards than those in the upper left. This is assumed due to the convergence location of the actor during training, though could be the manifestation of small asymmetries, such as fewer tracking errors in one half of the container.

These predicted rewards are tested through 1000 physical tests of the trained agent, randomized over the same state space. The measured reward distribution is plotted in Fig. 6’s lower row, where the predicted diagonal banded patterns are clearly visible. This reproduction of predicted behaviours suggests that the critic has learned a number of the physical system’s governing behaviours, using both successful and unsuccessful episodes to converge to a functional solution. As predicted, the actor performs best for radially-outward tasks, transitioning towards the lowest success rates for the $180^{\circ }$ tasks. The comparatively small effect of the starting radius comes in part from the elimination of extreme values for the starting positions (See *Control Space*), but also reflects the shaped reward of Fig. 4(b), which encourages the actor to rely on radially outward flows. Still, in both the measured and predicted cases, higher starting radii are associated with fractionally higher rewards; those which start closer to the end-effector are more prone to be affected by localised transients in the first seconds of motion, making the initial direction of the object less predictable. Additional quantitative results on the trajectory paths and reaching error from the 1000 physical tests of the trained agent are presented in Supplementary Figures S7, S8 and S9.

To systematically test the actor, 5 tasks (marked i–v) are chosen from Fig. 6, covering a range of starting and target positions. The tasks cover a range of predicted/measured successes: based on Fig. 6’s reward values, the agent appears to have developed partial or full solutions to Tasks i-iv, whilst Task v’s $180^{\circ }$ rotation is predicted an average reward of only 0.27. In the rest of this paper, we analyze and compare the solutions developed for each path and also try to explain why task v is more difficult to achieve.

**Figure 7:**
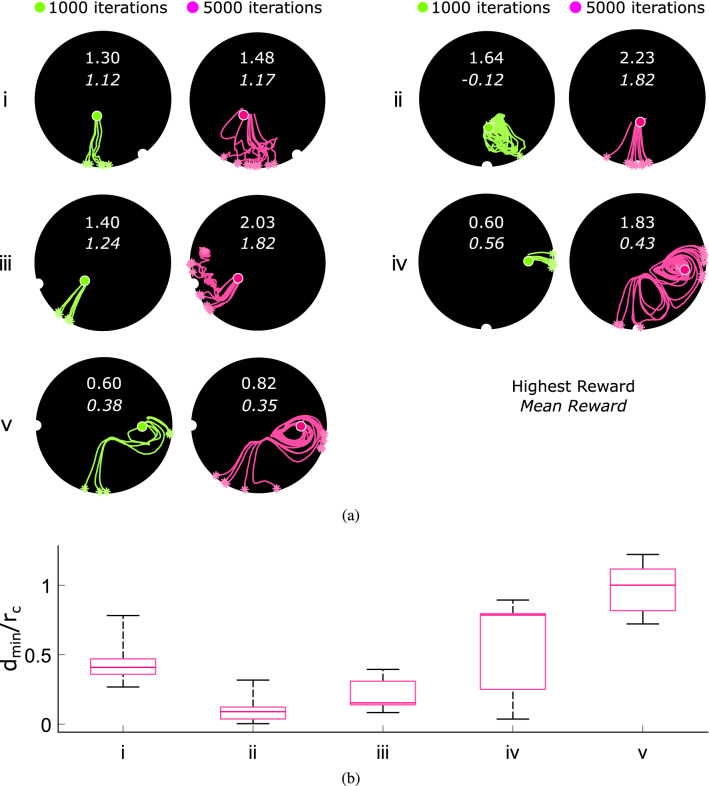
(**a**) Development of the agent’s solutions during training for the 5 selected tasks and their obtained rewards. Target locations are marked using notches on the container’s perimeter. (**b**) Box plots of d$_{min}$—the floating object’s minimum distance from the target—during 10 repetitions of each task, normalized using the container radius $r_c$.

Firstly, the trained agent’s proposed actions for each of the 5 tasks (Supplementary Tables S2 and S3) are performed 10 times, with the tracked paths shown in Fig. 7(a), and Supplementary Video 4. Five repetitions of the proposed actions after only 20% of Fig. 4(b)’s training are also plotted for the tasks, illustrating how the global controller’s behaviours develop over the final 4000 iterations of training. In all 5 tasks, the highest reward increases during these 4000 iterations, as more data becomes available. The reward shaping of Fig. 4 is apparent in Tasks i, iii, and iv: the agent learns to move the object radially outwards during the first 1000 training iterations before adjusting its trajectory to improve its reward. The mean reward increases in only 3 of the 5 cases: Tasks iv and v both demonstrate slight decreases, developing bifurcation points from which only one path leads to a high scoring outcome. This is why reward shaping is crucial in encouraging these high-risk-high-reward behaviors.

The increasing difficulty of Tasks ii-v is clear in Fig. 7(b), comparing the trained agent’s minimum distance to the target during the 10 repetitions. A satisfying solution to Task v is not developed—all other tasks demonstrate at least one path which passes within 50 mm of the target, with all of Task ii and iii’s repetitions passing within 72 mm. Small changes in initial position often cause the longer path of Task iv to prematurely reach the outer attractor—those which don’t exceed this critical point continue to highly rewarded solutions, explaining the larger range of Task iv’s solutions in Fig. 7(b). Overall, the trained agent is yet to reach the ability of the fixed Bayesian controllers presented in Fig. 3(b), suggesting that further training would lead to increasingly robust solutions.

Although all the tasks are repeated with the same starting position and control actions, the five tasks generate solutions with varying degrees of repeatability (Supplementary Figure S13b). Though our choice of control method assumes the underlying flow—and the resulting forces experienced by the floating object—to be fully deterministic, small amounts of stochasticity are still introduced into the experimental setup by imperfections and environmental noise. As such, control methods must rely on Lagrangian coherent structures to direct the object along the desired path and provide it with sufficient momentum to pass through more chaotic regions of the flow, as visualized in Supplementary Figures S11c and S11d. The increased length of the path developed to achieve this for Task iv increases the probability that these local stochastic perturbations about the Lagrangian structure will, at some point, be sufficient to exceed the separatrix with another attractor or mixing region. In Fig. 7(a), we can see that this occurs most frequently near the stable outer attractor, such that this region of the path acts as a bifurcation point during experimental repetitions of the task. This results in the trajectory of the controller being probabilistic for each run, even in the presence of unmodelled noise, justifying our Markovian assumption of the underlying dynamics (This is also confirmed by the Gaussian error distribution in Supplementary Figure S8).

**Figure 8:**
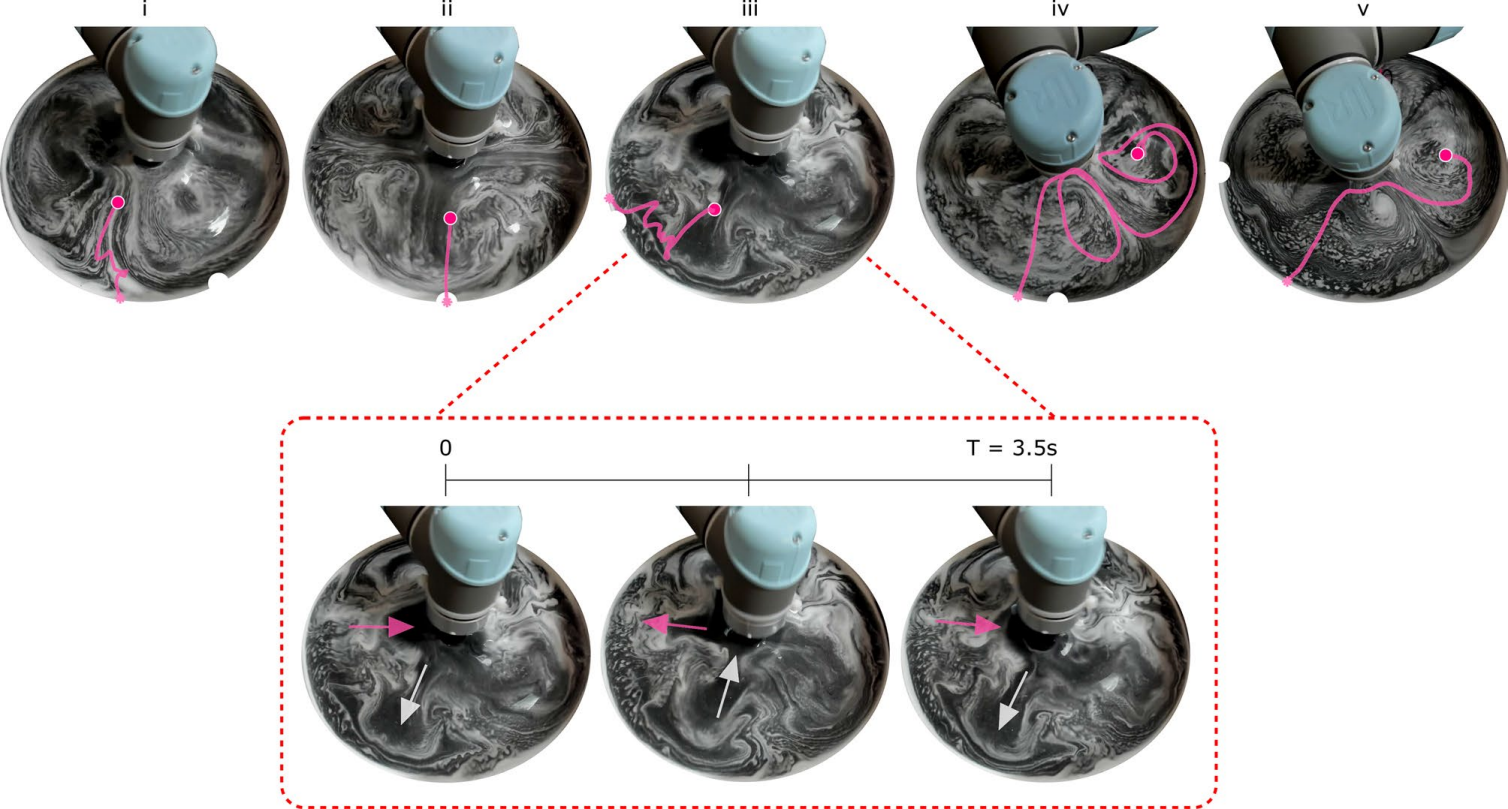
Visualizations of the surface flows generated for each of the five tasks using glass microsphere tracer particles, each overlain with their characteristic paths. The bottom row shows Task iii’s periodic flow pattern, with the directions of two major flow oscillations indicated with arrows.

Figure 8 uses K20 glass microspheres to visualize the generated flows of the 5 tasks: in iv and v, these flows appear to be steady, and the overlain trajectories follow the visible streamlines. Conversely, the flows of Tasks i-iii are quasi-periodic in nature, which can be seen in Supplementary Video 5. Their observed periods (73 s, 53 s, and 3.5 s respectively) can be estimated by assuming that the x and y oscillations dominate the controller’s behaviour:

1 $$\begin{aligned} T_{pred} = \frac{1}{|f_x - f_y|} \end{aligned}$$

giving predictions of 72.3 s, 53.0 s, and 3.4 s. The actions proposed for Tasks iv and v are both dominated by y oscillations, such that Eq. 1 gives expected periods <1 s, and the observed flows appear to be unchanging.

Task i’s paths experience, on average, 19% of a full period before completion (Supplementary Figure S12), The effect of this can be seen in Fig. 7(a)’s erratic nature: since the flow is not stationary in time, small temporal differences between repetitions lead to differences in the net force that the object experiences at a given point, thus affecting its final path. Task ii’s paths experience only 8% of a full period, and we see fewer such shifts in behaviour.

Conversely, Task iii’s average path lasts for 3.9 complete periods (Fig. 8), learning to use the periodic oscillations to develop a reliable control strategy, which can be seen in Supplementary Video 4. At $\hbox {t} = 0$ s, the object is pushed radially towards the outer attractor. If the capillary forces do not bring the object to the container’s edge, the flow then pulls the object inwards while moving the object clockwise towards the target. This process is repeated every 3.5 s until the stable outer attractor is reached. By using the whole period, this control strategy should transfer well to a variety of different object shapes—small delays in a new object’s path will not cause it to experience a wholly different flow.

To test this, we consider the effect of the object’s geometry on the trajectories experienced, evaluating the solution’s transferability without additional training. This is achieved by introducing 6 new shapes: 8 mm, 46 mm, and 72 mm circles, 22 mm and 35 mm squares, and a 25 mm quatrefoil (Supplementary Figure S5), which are each tested 5 times with Fig. 7(a)’s tasks and final actions.

**Figure 9:**
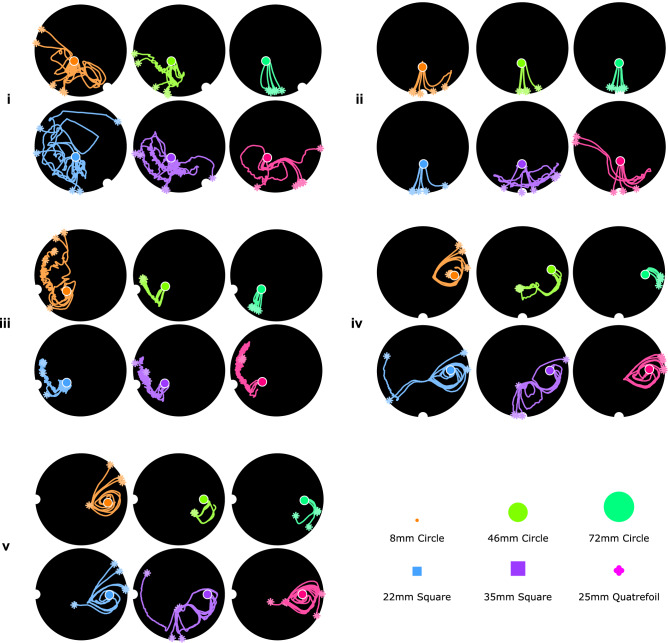
Transferability of the learned agent to a new set of shapes for the 5 example tasks. Each shape is tested 5 times without any additional training.

**Figure 10:**
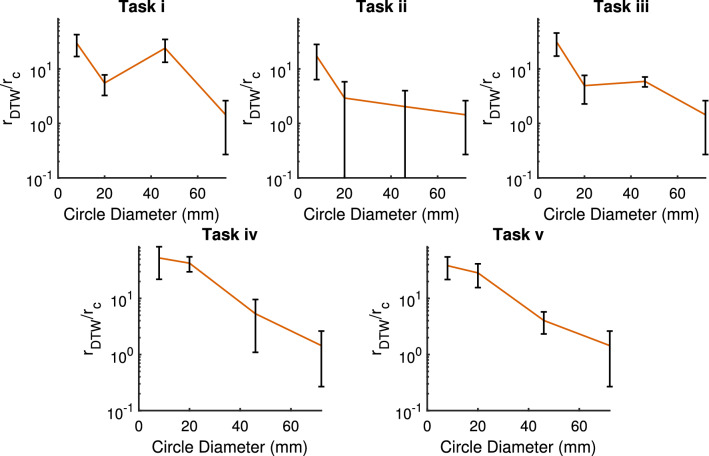
The difference in object trajectories, $r_{DTW}$, during repetitions of each task for different object diameters. A lower $r_{DTW}$ value indicates a highly repeatable trajectory, representing the minimized local cost measure during dynamic time warping. Values are normalized using the container radius $r_c$.

The paths followed by the six new objects are presented in Fig. 9. In all cases, it is noted that the surface flows generated by the oscillations are not significantly altered by the object geometry, with behavioural differences emerging from new interactions between the flow and the object. As expected, all of Task iii’s morphologies are brought close to the target via the periodic control strategy. However, the completion of the trained strategy relies on the separatrix with the outer attracter being reached after a small number of cycles, which is not always the case when the object is changed. Changes in circle diameter affect not only these behavioural boundaries, but the physical area which can be navigated; the 72 mm circle’s paths are dominated by early edge hits caused by the expansion of the outer attractor’s basin, whilst the 8 mm circle’s erratic behaviour is caused by its tendency to be caught in local surface flows. Figure 10 compares the repeatability of the trajectories for all circle diameters, demonstrating a correlation between circle diameter and trajectory similarity. As the area increases, the net force applied to the object by the underlying flow is less prone to be significantly affected by any small areas of non-stationary mixing, leading to smoother and more repeatable paths. The difference metric between every pair of trajectories, $r_{DTW}$, is calculated via dynamic time warping, with Euclidean distance between downsampled trajectories used as the local cost measure to be minimized (See *Experimental Setup*). The 20 mm $r_{DTW}$ values also reflect the task difficulties seen previously: Tasks iv and v, which require the largest angles to be traversed, show least similarity between repeated paths.

Clear correspondence to Fig. 7(a)’s paths can be seen throughout all tasks and morphologies of Fig. 9, suggesting that functioning controllers could be quickly adapted from another morphology’s agent as a starting point for training. The average rewards for each shape—given by Eqs. 6a and 6b—are presented in Supplementary Figure S10. Supplementary Section S2 presents an additional dimensional analysis of the problem geometries, where a clear dependence of the solutions on the container radius and invariance to the fluid depth is observed.

## Discussion

This study shows that deep learning techniques combined with large scale robotic experimentation can be used to develop effective controllers in the face of nonlinearities, non-convexity, and environmental stochasticity. We show not only that our agent learns to exploit Lagrangian coherent structures to produce optimal trajectories, but also that reward shaping based on the problem characteristics can be used to significantly reduce the learning period. Post-learning, a retrospective analysis of our solutions can be used to better understand the system’s complex underlying dynamics, with explorations of their dependence on environmental and geometric parameters aiding the development of increasingly robust controllers.

The proposed DRL architecture is highly desirable for this control task since unsuccessful trials can still be used to improve the critic and to aid exploration. Using insights from the physical setup, we further reduce the training time by reward shaping. We do this by encouraging outward radial motions in the early stages of training, avoiding non-repeatable behaviors. Fine tuning of the control policy then improves the overall reward. A high performing agent is obtained with our proposed methodology only after 5000 iterations. The total training time has upper bound of 3 minutes per iteration (up to 2 minutes for a full test plus 1 for the reset), giving an upper bound of 10.4 days of training for 5000 iterations. However, because of our reward’s shaping, the majority of attempts would hit the edge and stop early, thus taking less time. In the later stages of training, more than 1000 iterations could be averaged every day, thereby providing good solutions to the problem in reasonable times, a challenge with current physical experimentation platforms^14^. Multiple setups would permit parallel optimizations and further reduction in training time. This would also aid in finding policies that are more robust due to unavoidable errors incurred while replicating the setup^29^. Note that the number of iterations taken for the DDPG agent to learn a global controller is only an order higher than the iterations used by the Bayesian optimization for a fixed target and starting location. The DDPG algorithm combines the advantages of both value function learning and direct policy learning. The DDPG agent can utilize information from unsuccessful trials to update its internal models through the critic. Other reinforcement learning techniques like Q-learning can also be used for this problem, but we would need an additional search algorithm to get the optimal actions. Direct policy learning (like DDPG) is, however, better in many ways. Direct policy learning can exploit patterns between the optimal action for each state and hence be more efficient. It can also be used in continuous state and action space, which is not the case in traditional Q-learning. The disadvantage of DDPG is that it is difficult to train, unstable and highly sensitive to hyperparameters.

Techniques for object manipulation using localized energy sources would provide better controllability and accuracy over the proposed remote control strategy. However, remote manipulation is still the only available solution for certain applications. Manipulation by propagating waves are highly desirable in real-world scenarios where there are restrictions on the environment and target objects. On-surface and underwater robotic manipulation is the most straightforward bioinspired application, whilst current robotic technologies rely on direct contact with the target object^30–32^. The open-loop implementation greatly simplifies the sensory and on-board processing requirements. The transferability of the learned agent promises a practical approach to translate pre-learned controllers to changing environments and objects with minimal adaptation.

As we have framed the problem as an open-loop control problem, learning the control policy requires only simple sensory feedback. The only state information required for learning is the starting position of the object and the final error from the target. Hence, our setup can be replaced with ultrasonic or lidar sensors, thereby enabling us to scale up the application domain. A future work is to investigate the feasibility of this and to look into its possible application for waste debris and oil spill manipulation. Most notably, this would include explorations of the scalability of this method: Supplementary Section S2’s dimensional analysis has indicated that our solution depends on the dimensionless Laplace number, which varies linearly with the geometry. Further investigations are required to construct the relationships between the two, to determine how far from the end effector the object could feasibly be controlled. Another line of future investigation is to enable closed-loop control by providing state feedback of the floating object during manipulation. As the state information about the moving fluid is difficult to measure and estimate, we will therefore have to reformulate the problem as a Partially Observable Markov Decision Process. The disadvantages of this partial observability versus the advantages of having real-time feedback is to be investigated.

**Figure 11:**
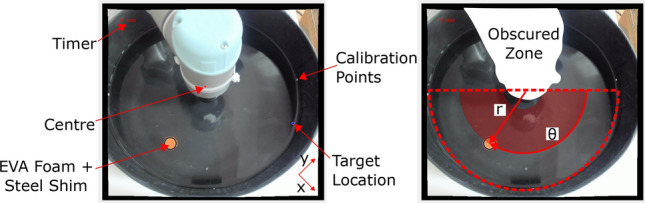
A typical view returned from the camera, indicating the obscured zone, the shaded area in which starting positions are randomized, and the operating semicircle in which target positions are set.

## Methods

### Experimental setup

The experimental setup consists of a tapered circular container filled with 8 L of water, with 85 mm water depth and 360 mm surface diameter (Fig. 1). Surface waves are generated from the container’s centre using a UR5 robotic arm equipped with a submersible end effector: a hollow PLA cylinder with 40 mm diameter and 80 mm length. An internal 12 V electromagnet is configured as a digital tool output of the arm such that, by holding the end effector above the water’s surface and powering the electromagnet, local magnetic objects on the water’s surface can be attracted and moved to a desired position. This forms the basis of the reset mechanism which allows the setup to autonomously run for extended periods of time (Supplementary Video 1 and Figure S4): the floating object is comprised of two layers of buoyant EVA foam sandwiching a circular steel shim, and can be retrieved and repositioned from any location in container using the electromagnet. To prevent the object from adhering to the end effector’s underside during this procedure, hot melt adhesive is used to texture its surface, limiting the contact area of the two.

To facilitate these resets, a webcam is positioned above the container, feeding live data to the PC which controls the robotic arm via Python: a typical view is shown in Fig. 11. The bright orange of the EVA object against the dark background of the container makes it straightforward to apply a binary mask and to identify the circular object using *MATLAB*’s image processing toolbox. Prior to this, the camera is calibrated to eliminate distortions in the water surface plane. Small white calibration points marked on the inside of the container are used to locate the surface’s centre and return locations in polar coordinates, where $\theta = 0$ is aligned with the camera’s horizontal orientation and the z direction is defined perpendicular to the plane of the water.

Figures 2 and 8’s flow visualizations are achieved by coating the surface of the water with a layer of K20 glass microspheres (Supplementary Video 6) before the vibrations begin. The webcam’s output is then fed into *MATLAB*’s estimateFlow function to create Fig. 2’s vector field. To create Fig. 10, the radial time-series of each trajectory is first interpolated/downsampled to contain 500 readings. Each pair of trajectories (i.e. for *n* repetitions, ${n \atopwithdelims ()2}$ pairs are considered) is fed into *MATLAB’s* dtw dynamic time warping function, which returns the minimised Euclidean distance between the stretched paths, $r_{DTW}$.

### Control space

The DDPG agent takes three inputs: the object’s starting position in 2D polar coordinates and the target periphery position, which is expressed using its polar angle. The ranges of the starting radius ($40 \rightarrow 100$ mm) and starting/target angles ($0^{\circ }\rightarrow 180^{\circ }$) are linearly normalized to the range (−1,1) throughout experimentation, marked by the shaded area in Fig. 11. This 3D state is sufficient to define the entirety of a single task, from which the agent must propose a suitable set of end-effector motions which obtain the highest reward.

These motions are defined using 11 control parameters, controlling orthogonal vibrations in the 3 Cartesian axes. Table 1 describes the 11 control parameters and their minimum/maximum limits. A sine wave’s mean (depth of submersion, *d*), amplitude, and frequency are defined in the z direction. The x and y axes are are each associated with a vibration frequency (*f*), amplitude (*A*), and phase ($\phi$), relative to the z vibrations), with zero mean. The position ${\mathbf {p}}$ of the end effector’s tip can then be described as:

2 $$\begin{aligned} {\mathbf {p}} = \begin{bmatrix} A_x\sin \left( 2\pi f_xt+\phi _x\right) &{} A_y\sin \left( 2\pi f_yt+\phi _y\right) &{} A_z\sin \left( 2\pi f_zt\right) - d \\ \end{bmatrix}^T \end{aligned}$$

where ${\mathbf {p}} = {\mathbf {0}}$ corresponds to the centre position at the surface of the water. The maximum values amplitude and frequency values (4 mm and 5 Hz, respectively) are chosen to avoid excessive vibrations of the table to which the arm is mounted, and to prevent water from spilling from the container edges. The maximum depth, *d*, is selected such that the arm is protected from water damage, with a minimum value ensuring that part of the end effector is always submerged during oscillations. The phases are not limited, and can take any value from $0\rightarrow 360\,^{\circ }$.

The end effector can also rotate about this point at its tip. To limit the number of control parameters such that the agent can converge to a global solution in a reasonable amount of time, these rotation values—$\theta _x$ and $\theta _y$—are defined as constant values throughout each iteration. Their maximum/minimum values are set to $\pm 10^{\circ }$, chosen to avoid interactions between the arm and container edge. Rotations about the z-axis are ignored, given the axisymmetry of the end effector. Like the inputs, the agent’s outputs—the 11 control parameters—are normalized to (−1,1).

**Table 1:** The 11 control variables which parameterise the end effector’s vibrations.

	${\text {A}}_{\mathrm{x}}$ (mm)	${\text {f}}_{\mathrm{x}}$ (Hz)	$\phi _{\mathrm{x}}$ (°)	$\theta _{\mathrm{x}}$(°)	${\text {A}}_{\mathrm{y}}$ (mm)	${\text {f}}_{\mathrm{y}}$ (Hz)	$\phi _{\mathrm{y}}$(°)	$\theta _{\mathrm{y}}$(°)	${\text {A}}_{\mathrm{z}}$ (mm)	${\text {f}}_{\mathrm{z}}$ (Hz)	${\text {d}}$ (mm)
Min.	0	0	0	−10	0	0	0	−10	0	0	5
Max.	4	5	360	10	4	5	360	10	4	5	45

To check these parameter values, we first consider the fluid in the immediate vicinity of the oscillating end effector: as motion begins, the water is stationary, and the flow is assumed similar to that of a two-dimensional flow around a smooth cylinder. At the maximum amplitude (4 mm) and frequency (5 Hz) of a one-dimensional vibration, the maximum Reynolds number is then:

3 $$\begin{aligned} Re = \frac{\rho uL}{\eta } = \frac{997\cdot 0.13\cdot 0.04}{0.00089} = 5.6 \times 10^3. \end{aligned}$$

This is comfortably beyond the idealization’s fully laminar range: vortices develop behind the cylinder, and the boundary layer begins to transition from laminar to turbulent at the highest amplitudes and frequencies. In addition, Supplementary Figures S11a, S11b, S11c and S11d visualize the forward and backward-time Finite Time Lyapunov Exponents (FTLE) calculated from a short video clip (1/6 seconds) of the microsphere-containing flows^33^. Though experimental noise prohibits exact measurement of the FTLE measurement over the flow, we can still observe how the surface flows affect the object trajectory. Regions where the FTLE is small are marked in white, regions of where activity propagates from (forward FTLE) and activity propagates towards (backward FTLE) are shown in orange and purple respectively. Regions of more predictable behaviour remain clearly visible in white, whilst densely populated regions—not just around the end effector—indicate regions of the underlying flow which are closer to chaotic behaviours.

The lower half of Fig. 1 depicts a typical view returned from the camera during the training stages. The interface displayed during each iteration marks the container’s centre, the object’s current position and target position, and displays the time elapsed since motion began. Given the development of an open-loop controller, this data is not fed back to the agent during each iteration, but is used after its completion to evaluate performance and allocate a suitable reward function. The only exceptions are the stopping conditions: an iteration is ended if the identified location reports that the object has hit the container edge, where capillary forces will retain it, or if two minutes have elapsed and the edge has not been reached. Before each iteration, a starting position and target location within Fig. 1’s operating region are randomly selected, and the object is positioned using the end effector’s electromagnet. The polar coordinate frame, common to the UR5 and vision system, is also marked.

### BO and DDPG implementation

The input states do not change between Bayesian iterations, with each optimization process focusing on developing the best 11 vibration parameters for one of the three rotation cases. Following the completion of each iteration, a cost function uses the tracked path to sum the distance from the target and the time elapsed:

4 $$\begin{aligned} f = l_{min} + t_f \end{aligned}$$

where $t_f$ indicates the time in seconds before either stopping criterion is fulfilled, and $l_{min}$ is the shortest distance recorded in mm between the object’s position and the target location over the whole trajectory.

The Bayesian optimization is implemented using *MATLAB*’s Statistics and Machine Learning toolbox. The three optimizations of Fig. 3(a) all use the same parameters (0.5 exploration ratio, Gaussian active set size of 300, and expected-improvement-per-second-plus acquisition function), and are halted after 680, 635, and 677 iterations respectively. The optimizations begin with no prior knowledge of the system’s setup or nonlinear dynamics, and only receive updates through the action-in/cost-out black box implementation.

For training of the DDPG agent, the naïve reward function focuses only on minimising the distance:

5 $$\begin{aligned} R\left( l_{min}\right) =3.45\left[ \exp \left( \frac{-l_{min}}{k_l}\right) \right] - 1.15 \end{aligned}$$

where $k_l = 165$ mm. A perfect hit of the target corresponds to a maximum reward of 2.3, decaying exponentially with $l_{min}$ towards a minimum of −1.15. The training is halted after 4151 iterations, when little improvement is seen in the controller.

The shaped reward function consists of the sum of two Gaussian functions and a small time dependence:

6a $$\begin{aligned} R\left( l_{min}, \Delta \theta , t_f\right) = \exp \left( -\frac{\Delta \theta ^2}{2{\sigma _\theta }^2}\right) + \exp \left( -\frac{{l_{min}}^2}{2{\sigma _d}^2}\right) - \frac{t_f}{k_t} + 0.5 \end{aligned}$$

in which $\sigma _\theta = {\pi }\big /{3}$, $\sigma _d = 50$ mm, and $k_t = 120$ s. $\Delta \theta$ is the angle between the target location and the object’s final location, allocated only if the positional stopping criterion is fulfilled. If the temporal stopping criterion has instead been fulfilled, then the first Gaussian function is replaced with a negative reward:

6b $$\begin{aligned} R\left( l_{min}, t_f\right) = -1 + \exp \left( -\frac{{l_{min}}^2}{2{\sigma _d}^2}\right) - \frac{t_f}{k_t} + 0.5 \end{aligned}$$

The combination of the reward described in 6a and 6b specifically discourages behaviours of the object which do not reach the container’s edge within the 2 minute time period (referred to as ‘uncompleted’ behaviours), whilst the function tends smoothly to a maximum of 2.25 amongst behaviours which reach the edge. The minimum reward tends to −1.25, for uncompleted behaviours which remain far from the target location throughout. Training is terminated after 5000 iterations.

The passing of the object into Fig. 1’s obscured zone is not explicitly discouraged by the cost and reward functions. Paths which pass through the zone and regain camera visibility are not penalised, but those which reach the edge in this region are considered uncompleted during the reward allocation. In this way, these paths pose a risk to the agent which does not exist when the zone is avoided. Coupled with the initialisation of all starting positions and target locations into Fig. 1’s operating semicircle, behaviours rarely rely on motion within this zone.

The trained DDPG agent is implemented using *MATLAB*’s Machine Learning toolbox, using two deep neural networks for its actor and critic architectures. Additional details, including an explanation of the chosen architecture, is given in Supplementary Section S1.

Between the actor’s 3-node input layer and its action space output, 5 hidden layers are implemented:

1. Fully connected (FC) layer with 50 nodes
2. Rectified linear unit (ReLU)
3. 50-node FC layer
4. ReLU
5. FC layer of same size as action space

When Reward Function b (Eqs. 6a and 6b) is used, this final layer has size 11 (Table 1), whilst Reward Function a (Eq. 5) uses a 12th decay parameter *D* (ranging from 2 to 502) to linearly reduce the amplitude parameters at each time step:

7 $$\begin{aligned} A_x(t) = \max \left[ 0, \left( 1-\frac{t}{D}\right) A_x(0)\right] ; A_y(t) = \max \left[ 0, \left( 1-\frac{t}{D}\right) A_y(0)\right] ; A_z(t) = \max \left[ 0, \left( 1-\frac{t}{D}\right) A_z(0)\right] \end{aligned}$$

The decay parameter was not used for the second agent as it did not improve the performance of the controller. The critic has two input layers, for the state and proposed action. Each is initially processed separately: the state is passed through a 50-node FC layer, ReLU and a second 50-node FC layer, whilst the action is fed into a single 50-node FC layer. An additional layer then combines the two, before a ReLU and FC layer reduce the output dimensionality to 1: the reward estimate.

## Supplementary Information

Supplementary Information 1.

Supplementary Information 2.

Supplementary Information 3.

Supplementary Information 4.

Supplementary Information 5.

Supplementary Information 6.

Supplementary Information 7.

Supplementary Information 8.

## Data Availability

The associated code & data may be found at https://github.com/DSHardman/FreeFloatingObjectsDDPG.
