# Supplementary material for: Manipulation of free-floating objects using Faraday flows and deep reinforcement learning
Source: Sci Rep. 2022 Jan 10;12:335. doi: 10.1038/s41598-021-04204-9 (PMC8748864; doi:10.1038/s41598-021-04204-9)
Supplement: Supplementary file 8 — Supplementary Information 8. [file 41598_2021_4204_MOESM8_ESM.pdf]

Manipulation of Free-Floating Objects using Faraday Flows and Deep Reinforcement Learning: Supplementary Material

S1 DDPG Implementation

DDPG uses an deep actor-critic architecture to learn control policies for continuous action spaces. DDPG is an off-policy reinforcement learning method that concurrently learns a value function and a policy. It uses off-policy data and the Bellman equation to learn the value function, and uses the value function to learn the policy. The DDPG agent obtains the actual reward from the real-world experiments after each episode and updates the actor and critic using a mini-batch of experiences randomly sampled from the experience buffer. To update the parameters of the actor and critic, random mini-batches of experience from the replay buffer are used. A small replay buffer will cause the algorithm to overfit and lead to instability in learning, while a large replay buffer will slow down the learning process. The target networks are time-delayed copies of their original networks that slowly track the learned networks. Using these target value networks greatly improves stability in learning[1].

The architecture of the DDPG agent is shown in Figure S1. The training of both reward functions uses a critic learn rate of 0.01, mini-batch size of 64, and 10^6 replay buffer length. Given that each of the training iterations contains only one step, no discount factor is necessary. A threshold of 10 is defined for the critic's gradient, above which L_2 scaling is applied. At chosen points during training (marked on Figure 4's x axes), the agent's noise parameters and actor learn rates are manually adjusted, based on current rates of convergence of the actor and critic. Actor learn rates are initialised to 0.001, and increased to 0.002 after 3111 & 1946 iterations in Reward Functions 1 & 2 respectively. Similarly, both noise variances are initialised to 0.3: in Figure 4a, this is reduced to 0.15 after 2383 iterations and returned to 0.3 after 3111. In Figure 4b, it is reduced to 0.26 after 1448 iterations and allowed to decay to 0.12. The variance is adjusted to the constant values of 0.15 and 0.1 after 1946 & 2481 iterations respectively.

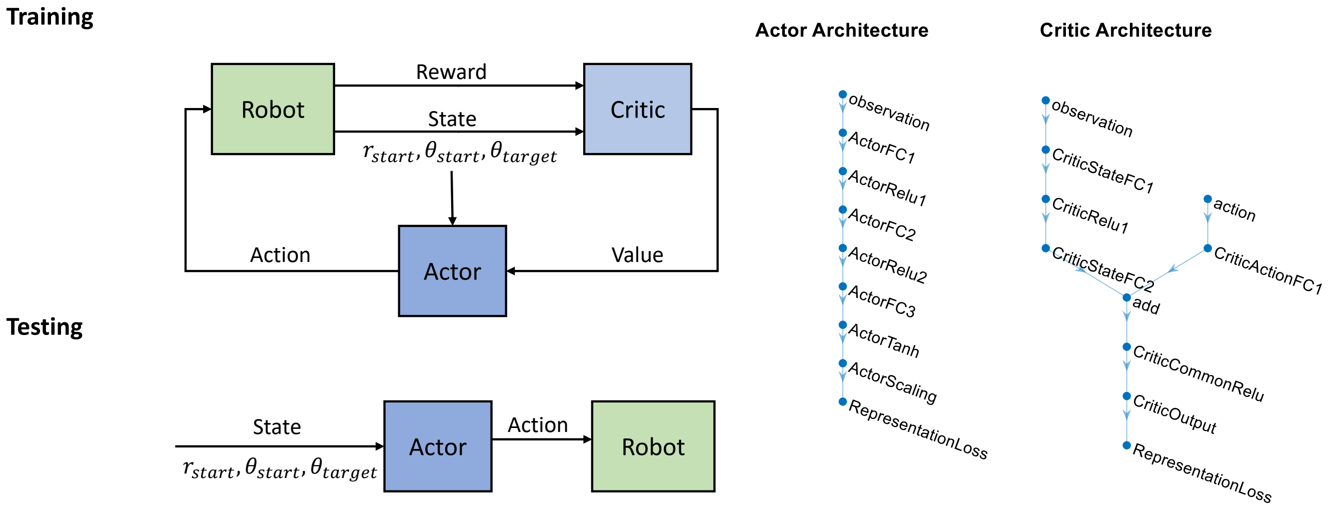

Figure S1. Left Top: The interactions between robot and DDPG agent - comprised of an actor and critic - during training stages. Left Bottom: The interactions between actor and robot during testing of the DDPG agent in Figures 5 to 9. Right: The deep neural network architectures of the actor and critic implemented in the DDPG agent.

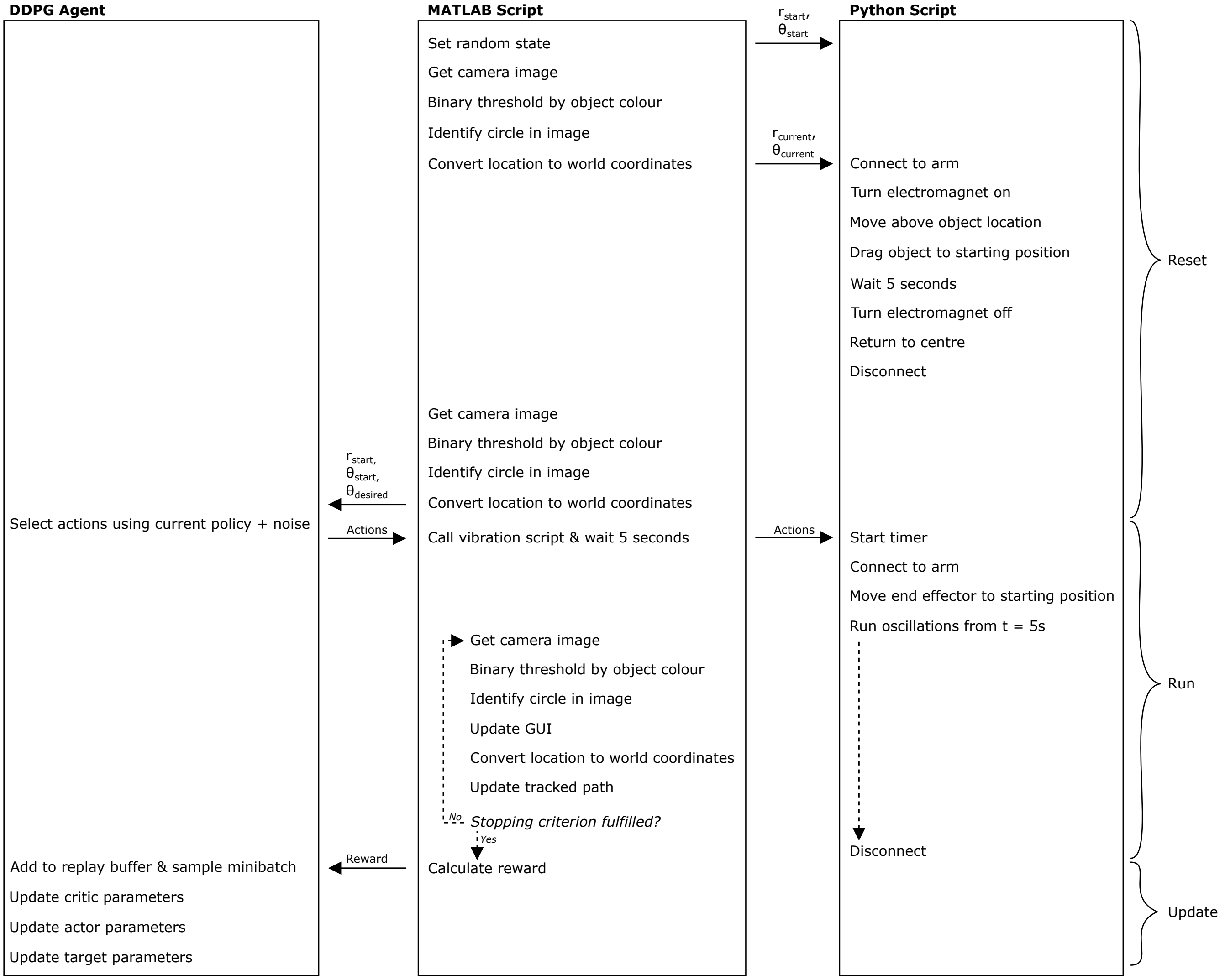

Figure S2. The steps involved in a single iteration of reinforcement learning. The process is controlled using a central MATLAB script which communicates with the DDPG agent, camera, and robotic arm's Python controller.

S2 Dimensional Analysis

Assuming the dimensionless reward function R to ultimately be a function of its 4 scaling parameters, the 11 action parameters, 3 state parameters, the problem geometry (container radius r_c, water depth h_c, end effector radius r_e, and floating object radius r_o), and the physical properties (g, and a fluid with density ρ, dynamic viscosity η, surface tension γ, and speed of sound c), an application of the Buckingham π theorem finds a dependency on 24 dimensionless groups:

$$R = \Pi\left(\frac{c^2\rho\gamma}{g\eta^2},\ \frac{c\eta}{\gamma},\ \frac{\gamma\rho}{\eta^2}r_e,\ \frac{c}{g}f_x,\ \frac{h_c}{r_e},\ \frac{r_c}{r_e},\ \frac{r_o}{r_c},\ \theta_{start},\ \frac{r_{start}}{r_c},\ \theta_{target},\ \frac{A_x}{r_e},\ \phi_x,\ \theta_x,\ \frac{A_y}{r_e},\ \phi_y,\ \theta_y,\ \frac{f_y}{f_x},\ \frac{A_z}{r_e},\ \frac{f_z}{f_x},\ \frac{d}{h_c},\ \frac{k_l}{r_e},\ k_t f_x,\ \sigma_\theta,\ \frac{\sigma_d}{r_e}\right) \tag{1}$$

of which 15 were varied during our experiments. For a situation in which these 15 are fixed, and the 4 scaling factors are held constant, the function becomes

$$R = \Pi\left(\frac{c^2\rho\gamma}{g\eta^2},\ \frac{c\eta}{\gamma},\ \frac{\gamma\rho}{\eta^2}r_e,\ \frac{c}{g}f_x,\ \frac{h_c}{r_e},\ \frac{r_c}{r_e}\right). \tag{2}$$

By focusing only on behaviours in water, and taking gravitational acceleration to be constant, we further simplify this to

$$R = \Pi\left(\frac{\gamma\rho}{\eta^2}r_e,\ \frac{h_c}{r_e},\ \frac{r_c}{r_e}\right) \tag{3}$$

i.e. the dimensionless reward is dependent on the geometric ratios $\frac{h_c}{r_e}$ & $\frac{r_c}{r_e}$, and on the Laplace number $\frac{\gamma\rho}{\eta^2}r_e$ which relates the fluid's surface tension and inertial forces. With constant fluid parameters, this is affected only by r_e. To investigate their effects on the generated Faraday flows, we perturb each of the dimensionless groups in turn (Table S1) by appropriately varying the water depth, container size, and end effector radius. A container with r_c = 94mm is used for perturbations A & B: it is assumed that the slight difference in the containers' taper and stiffness will negligibly affect the behaviours relative to the three independent groups. To maintain the radii ratio, a end effector with 10.4mm radius is used during perturbation B. The non-dimensional action parameters corresponding to Task iv's steady surface flow (Supplementary Video 3) are kept constant, and the resulting flow patterns are observed. These are presented in Figure S3 and in Supplementary Video 7. We see that

Table S1. Perturbations of the three independent non-dimensional groups from the original problem geometry.

	$\frac{\gamma\rho}{\eta^2}\mathbf{r_e}$ ($\times 10^6$)	$\mathbf{\frac{h_c}{r_e}}$	$\mathbf{\frac{r_c}{r_e}}$
**Original**	1.8	4.3	9.0
**Perturbation A**	1.8	4.3	**4.7**
**Perturbation B**	**0.95**	4.3	9.0
**Perturbation C**	1.8	**2.2**	9.0
**Perturbation D**	1.8	**6.0**	9.0

perturbations of approximately -50% of the groups $\frac{\gamma\rho}{\eta^2}r_e$ & $\frac{r_c}{r_e}$ (A & B respectively) do not maintain the original steady flow pattern, whilst similar patterns can clearly be seen in perturbations C & D when $\frac{h_c}{r_e}$ is varied $\pm$ 50%. If further investigations were to confirm that the floating object's path is relatively insensitive to this parameter, then small changes in water depth would not need to be considered during the agent's training, and the reward could instead be locally considered a function of two dimensionless groups for a given state/action:

$$R = \Pi\left(\frac{\gamma\rho}{\eta^2}r_e,\ \frac{r_c}{r_e}\right). \tag{4}$$

Though the generated flows are not independent of the Laplace number and radii ratio, we hypothesise that the same DDPG implementation could learn to control the systems described by perturbations A & B. Indeed, Supplementary Video 7 shows flow behaviours similar to those seen during the early stages of training in the original, suggesting that a re-trained agent would learn to similarly exploit the dynamics of the new system. In this way, wide ranges of problem geometry could be controlled.

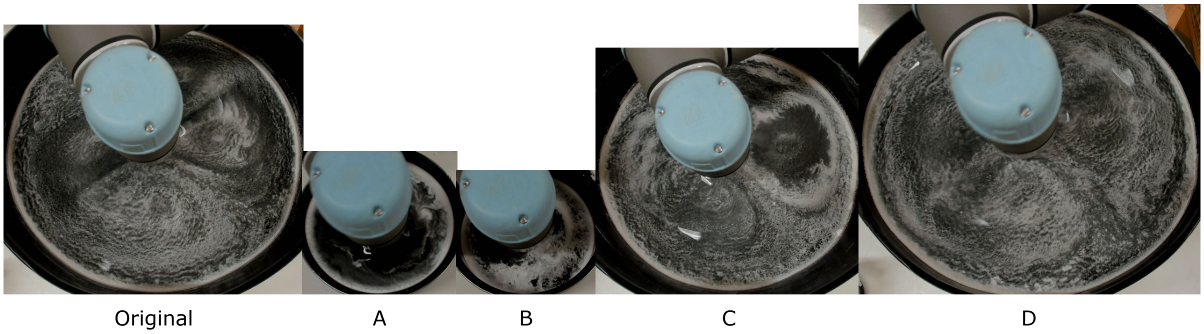

Figure S3. The surface flows generated by the four perturbations of the non-dimensional groups. A & B do not preserve the steady flow pattern, whilst C & D clearly show similar behaviours.

Supplementary Figures

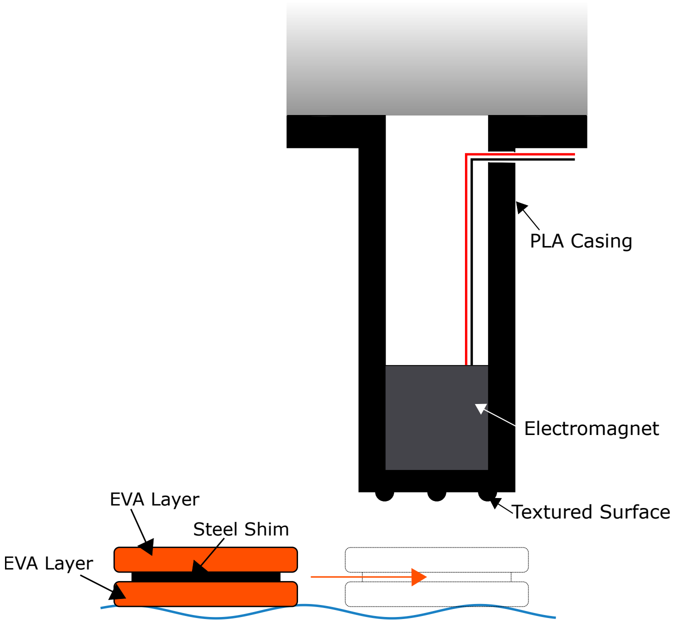

Figure S4. Cross sections of the 3D printed end effector and floating object used in the experiments (not to scale). When the robotic arm's tool output provides 12V to the electromagnet, the steel shim in the floating object draws the object underneath the end effector, where it can be dragged along the fluid surface to its starting position before the voltage is removed. The textured surface prevents the wet EVA foam from adhering to the underside of the end effector.

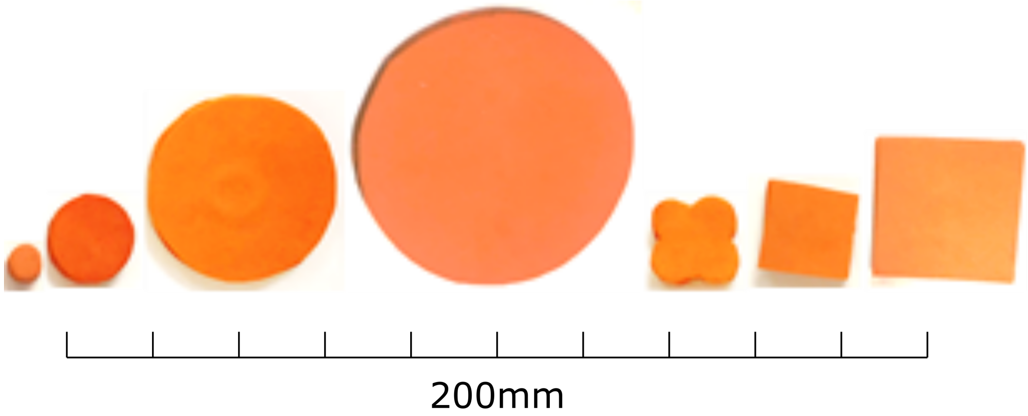

Figure S5. The seven floating objects. From left to right: 8mm circle, 20mm circle, 46mm circle, 72mm circle, 25mm quatrefoil, 22mm square, & 35mm square. The 20mm circle was used throughout experiments unless otherwise stated.

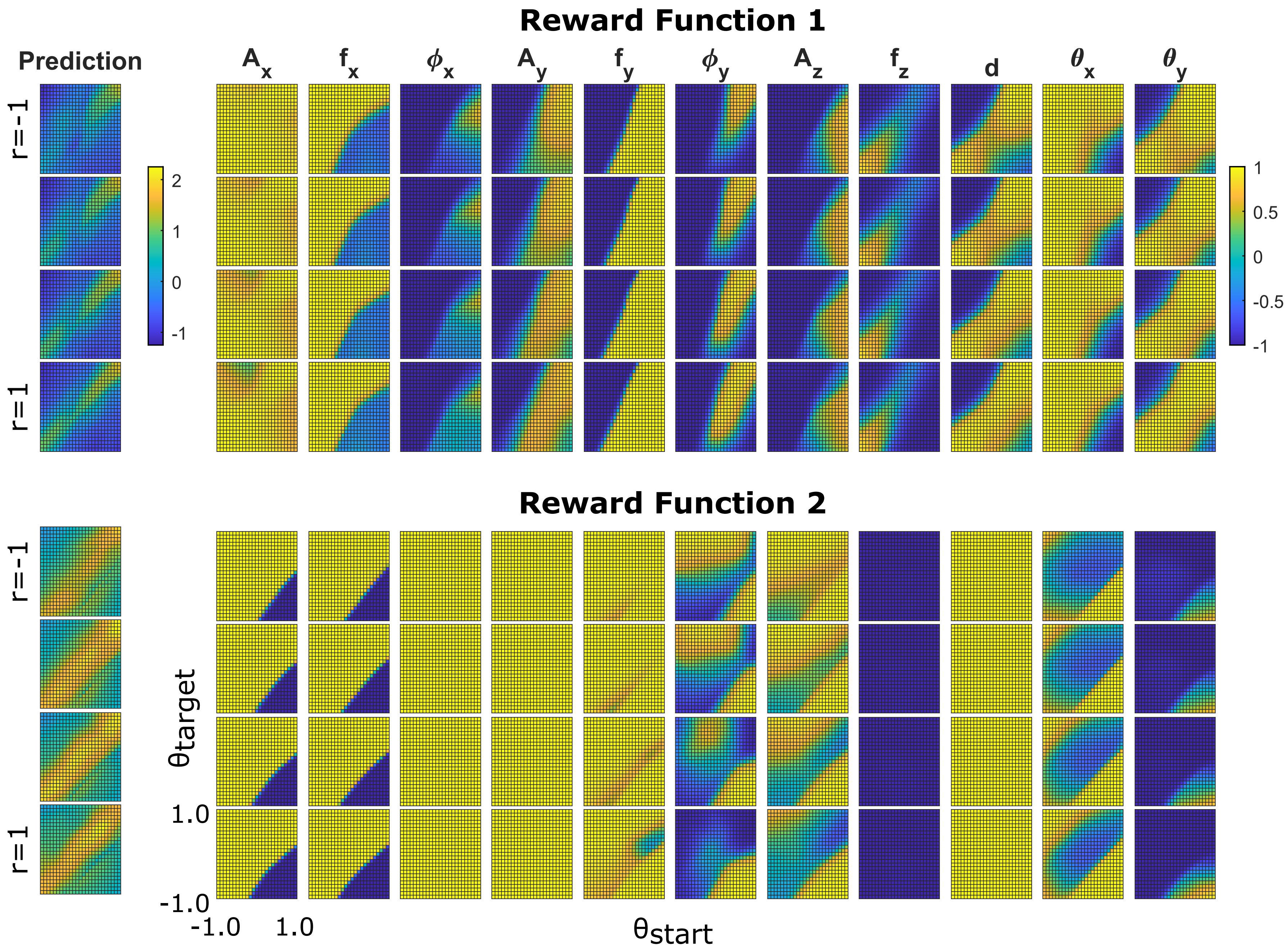

Figure S6. The complete action space of the agents trained using both reward functions, normalized in the range (-1,1). Each heatmap plots the proposed value of a single control variable over the full ranges of θ_{start} & θ_{target} whilst r_{start} is held constant. All actions use the same colour scale, whilst predictions use a separate (-1.25 2.25) range.

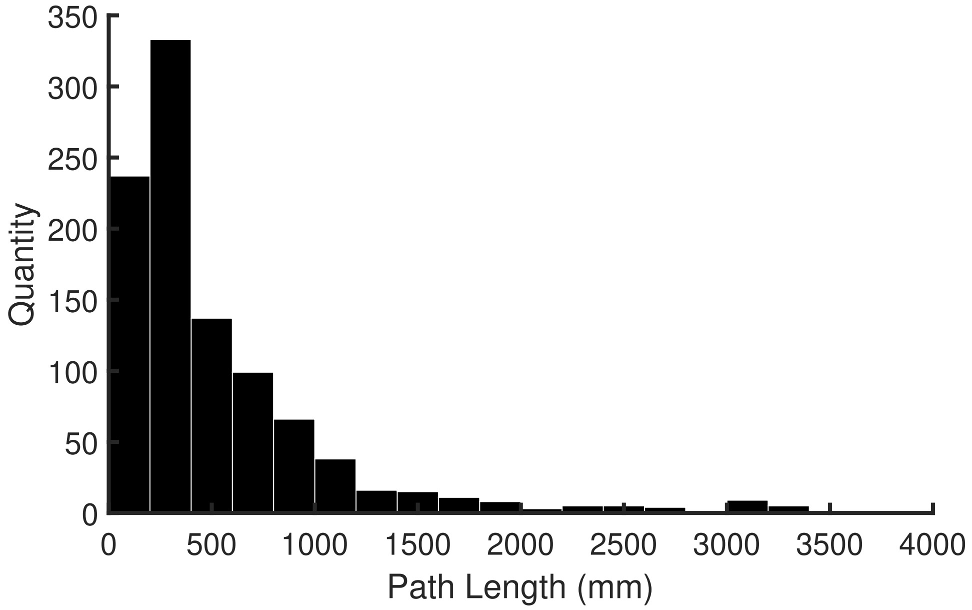

Figure S7. The distribution of total path length in mm over the trained agent's 1000 tests which comprise the bottom row of Figure 6. These are calculated using the sum of the Cartesian distances between all reported object locations over the tracked period. As such, the values are sensitive to small amounts of noise and variances in the camera's frame rate, and act as an upper bound of the genuine lengths.

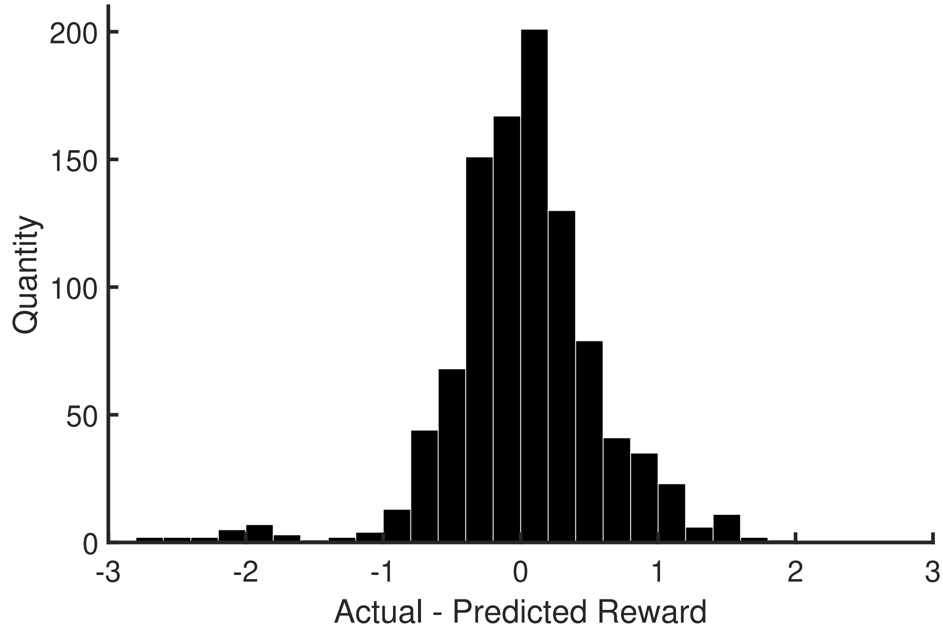

Figure S8. The distribution of errors (actual reward - predicted reward) over the trained agent's 1000 tests which comprise the bottom row of Figure 6.

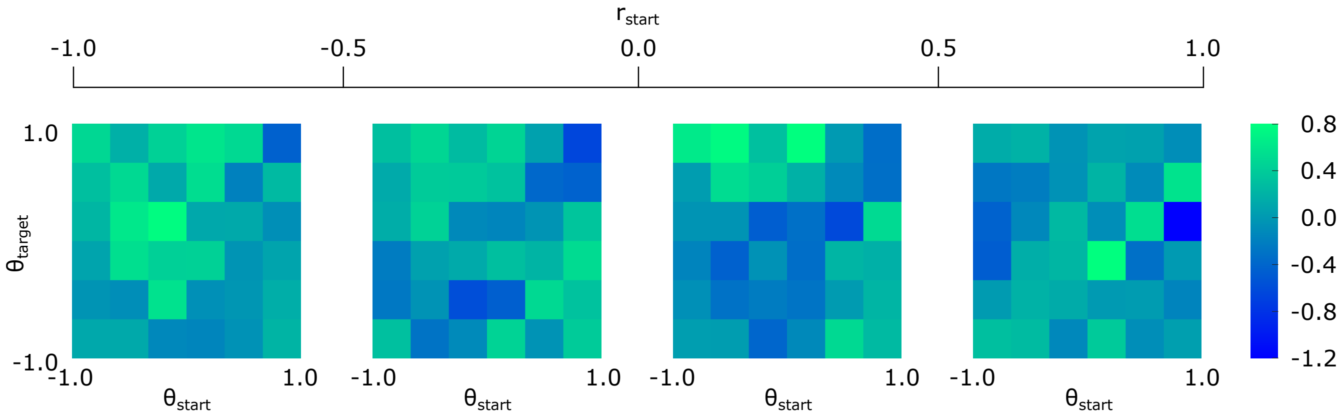

Figure S9. The location of errors (actual reward - predicted reward) over the trained agent's 1000 tests which comprise the bottom row of Figure 6. Note that the colour scale is *not* the same as that used in Figure 6.

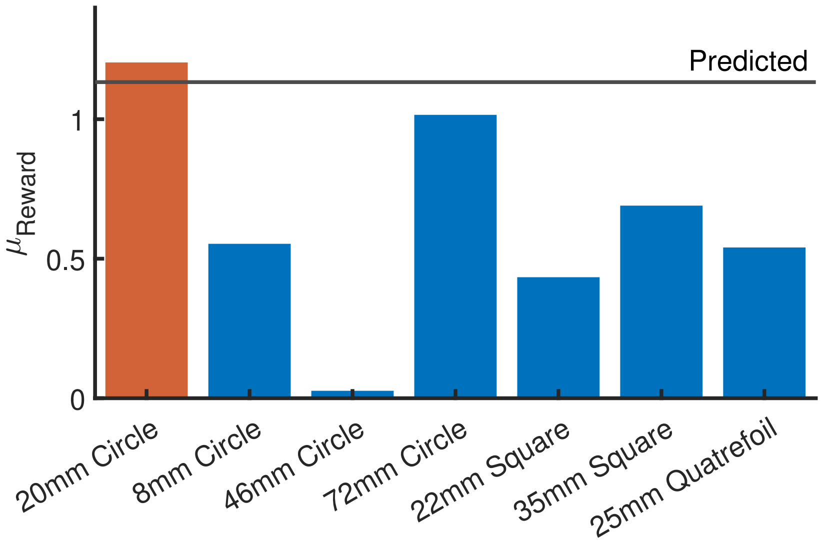

Figure S10. The average reward obtained by all shapes of floating object over the 5 tasks of Figure 9. The predicted average for the 20mm circle on which the agent was trained (1.13) is marked.

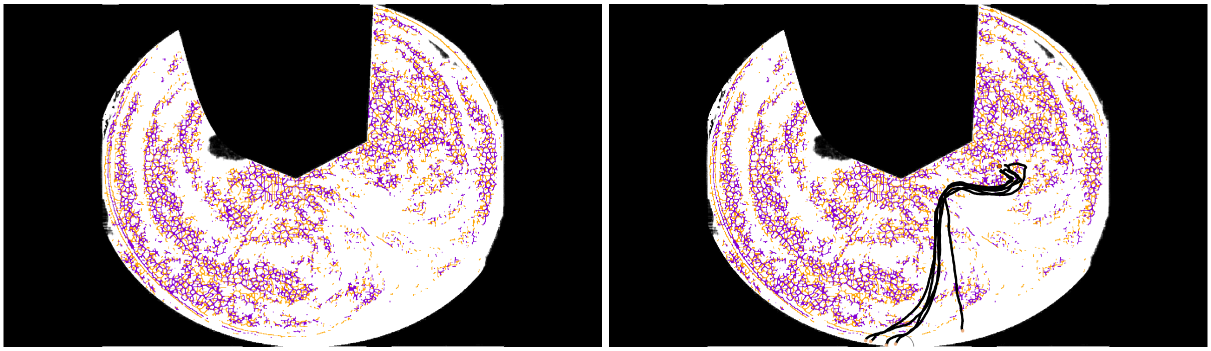

(a)

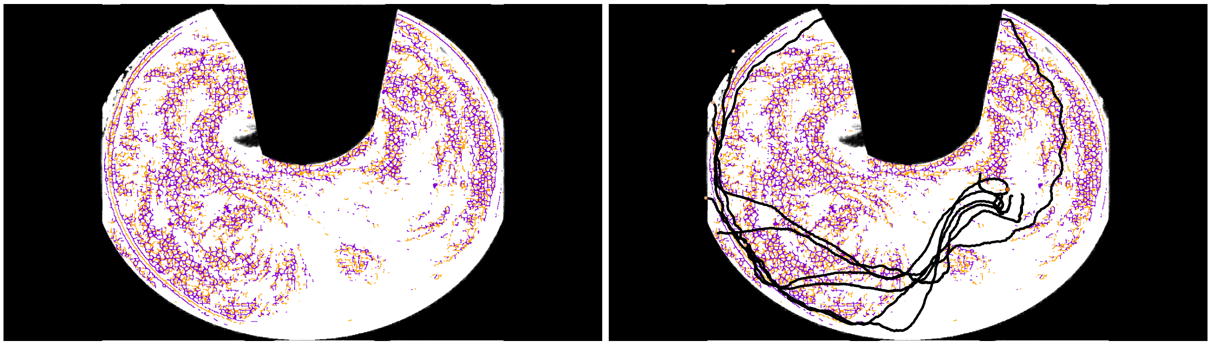

(b)

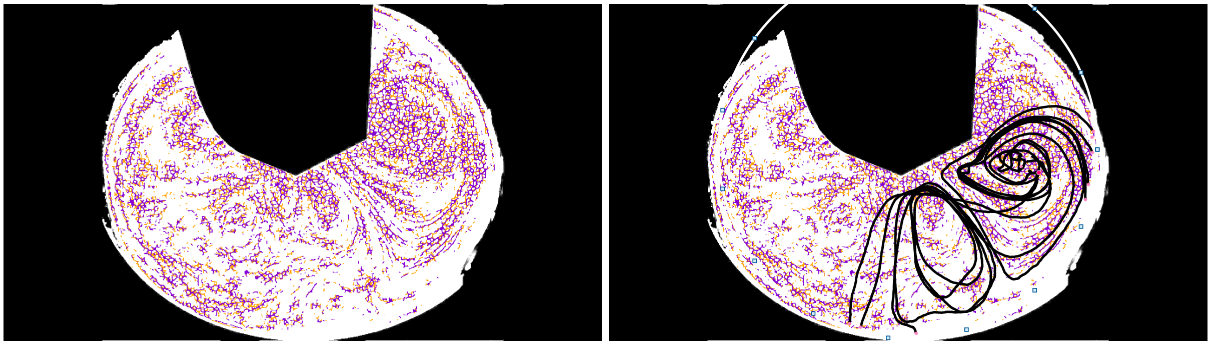

(c)

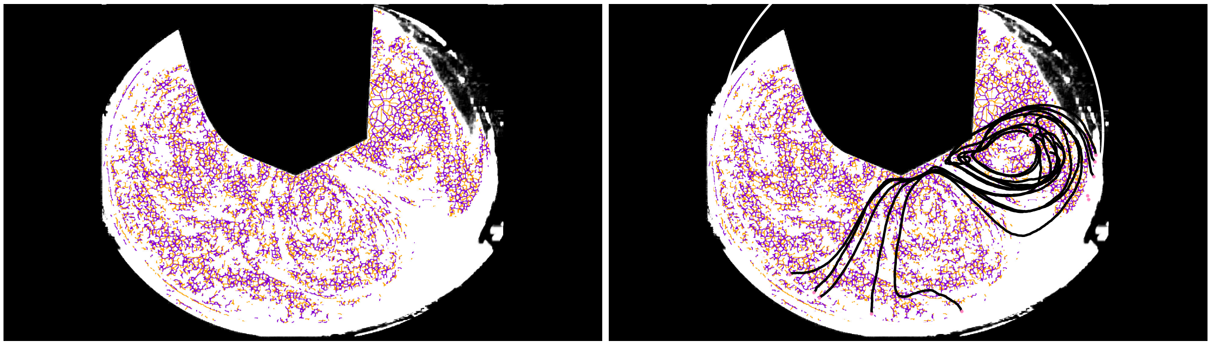

(d)

Figure S11. Visualizations of the forward and backward-time Finite Time Lyapunov Exponents (FTLE), taken from successive frames of Supplementary Videos 5 & 7. Lagrangian coherent attractors are identifiable as sparse areas[2]. (a) The 90° Bayesian trajectories. (b) The 180° Bayesian trajectories. (c) Task iv's trajectories. (d) Task v's trajectories.

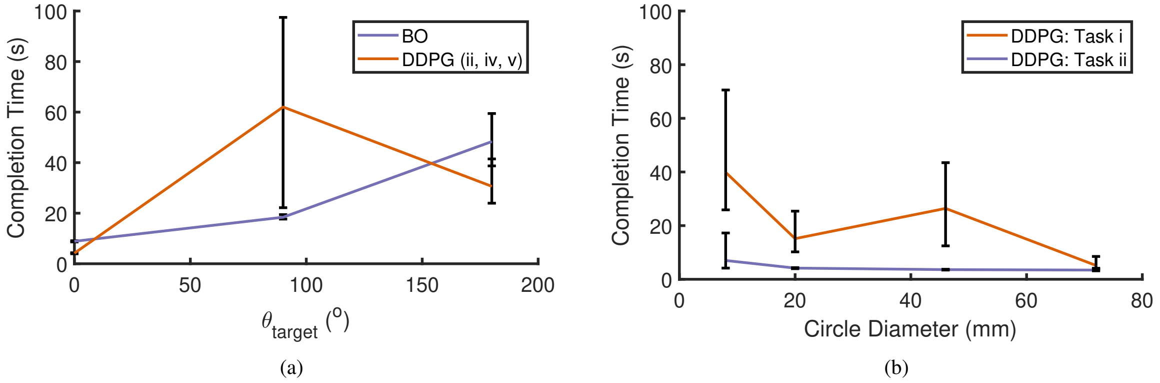

Figure S12. The times taken for successful completion of a trajectory. (a) With $\theta_{start} = 0°$, the time taken to rotate 0°, 90°, & 180° before reaching the outer attractor. (b) Tasks i & ii, tested on 4 different object sizes. The DDPG agent is trained using the 20mm circle.

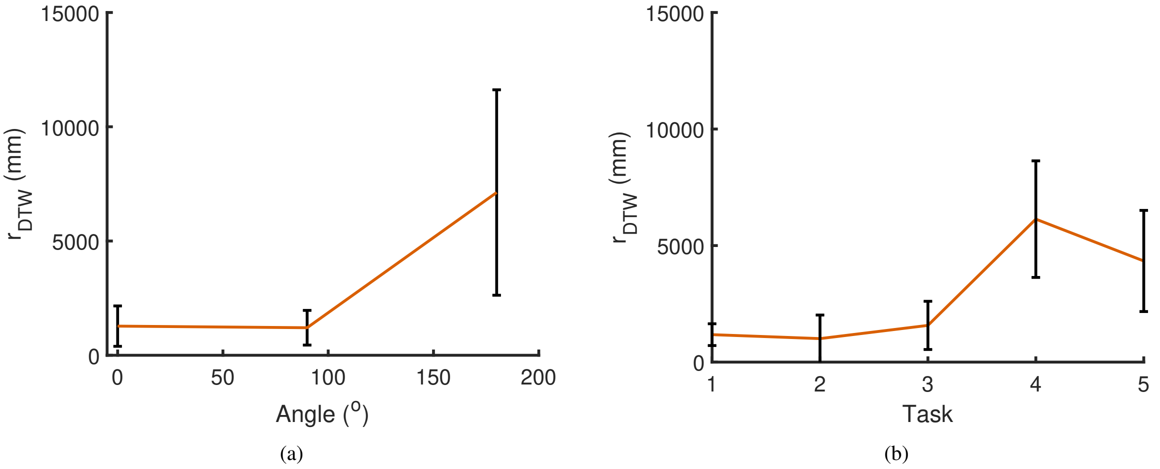

Figure S13. The difference in object trajectories, r_{DTW}, during repetitions of the Bayesian & DDPG tasks. r_{DTW} is calculated using dynamic time warping to match each pair of 500-sample radial time series, using Euclidean distance as the local cost measure. A lower r_{DTW} value indicates a highly repeatable trajectory.

Supplementary Tables

Table S2. The states which define each of the Tasks i-v, normalized in the range (-1, 1). These are selected to cover a wide range of the three dimensional state space, and correspond to the locations marked on Figure 6's top row.

	r_{start}	θ_{start}	θ_{target}
**Task i**	$-2/3$	0.4	-0.4
**Task ii**	$-1/3$	0.0	0.0
**Task iii**	$1/3$	0.5	0.8
**Task iv**	$2/3$	-1.0	0.0
**Task v**	$2/3$	-1.0	1.0

Table S3. The actions proposed by Reward Function 2's 5000th agent, normalized in the range (-1,1). These are the actions illustrated in Figures 7, 8, & 9. The corresponding minimum & maximum values for each of the variables are given in Table 1.

	A_x	f_x	ϕ_x	θ_x	A_y	f_y	ϕ_y	θ_y	A_z	f_z	d
**Task i**	1.00	0.99	1.00	-0.34	1.00	1.00	0.67	-0.95	0.91	-1.00	1.00
**Task ii**	1.00	1.00	1.00	-0.62	1.00	0.99	0.01	-0.94	0.39	-0.98	1.00
**Task iii**	1.00	1.00	1.00	-0.55	1.00	0.88	-0.89	-1.00	-0.25	-1.00	1.00
**Task iv**	-0.98	-1.00	0.99	1.00	1.00	1.00	0.72	0.32	0.94	-0.98	1.00
**Task v**	-1.00	-1.00	1.00	1.00	1.00	1.00	1.00	0.82	1.00	-1.00	1.00

Code & Results

The associated code & results may be found at https://github.com/DSHardman/FreeFloatingObjectsDDPG

Description of Supplementary Videos

Supplementary Video 1 is a timelapse of the robotic arm during the reinforcement learning process, containing 9 iterations and 8 resets. After a stopping criterion has been fulfilled, the floating object is reset to a new randomized starting position using the electromagnet.

Supplementary Video 2 shows the paths taken by the floating object using the final actions of the three Bayesian optimisations: 0°, 90°, & 180°. Each is repeated multiple times to demonstrate the solutions' level of repeatability. The flow patterns of the 0° & 180° cases are visualized using glass microsphere tracer particles.

Supplementary Video 3 depicts the visualized flows generated by the 0°, 90°, & 180° Bayesian solutions, played at 8× speed. The 0° flow is periodic, whilst the 90° & 180° flows are stable.

Supplementary Video 4 shows the paths taken by the floating object using the actions proposed by the trained agent for the Tasks i-v. Each is repeated multiple times and the most dissimilar behaviours included; the frequency with which each occurs is not represented in the video. The flow patterns are visualized using glass microsphere tracer particles.

Supplementary Video 5 depicts the visualized flows generated by trained agent for Tasks i-v, played at 8× speed. The periodicity of Tasks i & ii is illustrated by comparing the recordings with relevant time offsets, whilst just one recording is necessary to fully demonstrate the behaviours of Tasks iii-v.

Supplementary Video 6 contains the methodology of flow visualization. K20 glass microspheres are first added to the container at 8× speed to reveal the surface flow behaviours, before a sample of the generated flow pattern is overlain onto its source footage.

Supplementary Video 7 contains the visualized flows from the four perturbations introduced in the Dimensional Analysis section. A & B do not preserve the steady flow, whilst C & D are clearly similar to the original.

References

1. Lillicrap, T. P. *et al.* Continuous control with deep reinforcement learning. *arXiv preprint arXiv:1509.02971* (2015).

2. Linden, N. J. *et al.* Go with the flow: Visualizing spatiotemporal dynamics in optical widefield calcium imaging. (2020).
